# Complexity and Entropy Analysis of a Multi-Channel Supply Chain Considering Channel Cooperation and Service

**DOI:** 10.3390/e20120970

**Published:** 2018-12-14

**Authors:** Qiuxiang Li, Xingli Chen, Yimin Huang

**Affiliations:** 1 Institute of Management Science and Engineering, Henan University, Kaifeng 475004, China; 2 College of Business, Henan University, Kaifeng 475004, China; 3 College of Management & Economics, North China University of Water Resources and Electric Power, Zhengzhou 450046, China

**Keywords:** multi-channel supply chain, cooperation, service, chaos, entropy

## Abstract

In this paper, based on the background of channel cooperation and service of the supply chain, this paper constructs a Nash game model and a Stackeberg game model in the multi-channel supply chain considering an online-to-store channel (OSC). Based on maximizing the profits and the bounded rationality expectation rule (BRE), this paper builds a dynamic game model, respectively, and analyzes the stability of the equilibrium points by mathematical analysis and explores the influences of parameters on stability domain and entropy of the system by using bifurcation diagram, the entropy diagram, the largest Lyapunov exponent and the chaotic attractor etc. Besides, the influences of service level and profit distribution rate on system’s profit are discussed. The theoretical results show that the greater the service level and profit distribution rate are, the smaller the stability domain of the system is; the system will go into chaotic state and the system’s entropy will increase when operators adjust her/his price decision quickly; when the manufacturer or the retailer keeps service level in the appropriate value which is conducive to maximizing her/his profit; the manufacturer should carefully set the service level of OSC to ensure the system’s profit; in Nash game model, the stability of the system weakens than that in Stackelberg game model. Furthermore, this paper puts forward some suggestions to help the manufacturer and retailer in multi-channel supply chain to do the better decision.

## 1. Introduction

According to the data from a Chinese 2017 e-commerce development report, the scale of e-commerce transactions in China continued to expand in 2017 and maintained rapid growth [1], but with the rapid development of e-commerce, mobile commerce and the changing of customer’s demands, more and more enterprises such as Jing Dong, Tmall and Uniqlo are beginning to participate in the innovation of retail channels based on online channel and traditional channel. Many retailers are now allowing their customers to pick up online orders at physical stores (channel cooperation), which is revolutionizing retail operations to some extent. Multi-channel sales can help companies to improve the efficiency of supply chain management and market share.

A lot of scholars had done extensive and in-depth research on multi-channel supply chain [2,3,4]. Breugelmans and Campo [5] examined the cross-channel effects of price promotions on category purchase decisions, and showed that promotions in one channel can have negative effects on category purchases in the other channel during the promotion period. Matsui [6] investigated the optimal timing and level of wholesale and retail prices set in multi-channel supply chains, where a manufacturer produced and sold products to retailers that compete to resell the products by applying the framework of an observable delay game devised in no cooperative game theory. In a multi-channel supply chain with the manufacturer’s online direct channel and the hybrid retailer’s online and offline channels, Yu et al. [7] analyzed the optimal wholesale price of the manufacturer and retailer when they centralized control and decentralized control with Stackelberg game theory separately, and the result showed that decentralized control can lower the efficiency of the supply chain. Esmaeilzadeh and Taleizadeh [8] aimed at exploring the optimal pricing decision of two complementary products in the two level supply chains under different market forces. Li et al. [9] constructed the pricing model of the multi-channel supply chain under the competition of the two brands. Dan et al. [10] constructed a Stackelberg price game model with the strong retailer as the leader, studied the pricing decision process of the supply chain members and analyzed the influence of key channel factors on the scale of the market such as electric channel market scale expansion and dispersible percentage of electric channels. Ma and Lou [11] studied the complexity of the price competition in the multi-channel household appliance supply chain and found that the system in chaotic state would be more volatile than that in stable state, and the manufacturer’s network channel helped to alleviate the effect of the bullwhip effect. In addition to the study of price strategy, some scholars have studied the problem of inventory and return in multi-channel supply chain [12,13,14]. Hubner et al. [15] analyzed the different multi-channel networks and the associated inventory management approaches as well as the various design concepts in warehouse operations, taking into account both contemporaneous effects (during the promotion period) and cross-period effects (after the promotion period). Luo et al. [16], considering the false failure returns and consumers’ heterogeneous channel preferences, examined the hybrid retailer’s channel integration strategy by offering e-coupons to attract consumers to try out and purchase products in the physical store.

The above papers studied the price strategy in the multi-channel supply chain considering different situations which provide a reference for managers to make a decision. However, the above papers were confined to the price study of multi-channel supply chain, didn’t involve the research of price and service simultaneously.

With the development of market economy and the change of consumer demand, the impact of channel service on multi-channel supply chain has also become the focus of attention in business and academia. The multi-channel supply chain improves the actual demand by providing better services to customers. In general, such services include delivery, replacement, maintenance, warranty, after-sale support, regular updates and other services that can increase customer perception of product value. Service has become an important factor affecting consumer’s channel choice and profit in a supply chain [17]. Ren et al. [18] found the cost of the manufacturer and the network retailer will be increased by allowing the customer to return, and the sales price of the product will be affected. Pei and Yan [19] proposed supportive retail services as an effective incentive to coordinate the dual-channel distribution and govern the relationship between channel members. Li and Li [20] designed the dual- channel supply chain; thus channel competition became in evitable. Besides, value-added services provided by retailers were considered. Equilibrium problems regarding retail service and fairness concerns were analyzed. Protopappa-Sieke et al. [21] developed the optimal two-period inventory allocation policies under multiple service level contracts in view of the fact that optimal inventory allocation had a significant impact on profits in the retail industry. Zhou et al. [22] investigated how free riding affects the two members’ pricing/service strategies and profits when the dual channels use the differential and non-differential pricing scenarios, respectively. Sadjadi [23] introduced the retailer Stackelberg game; two manufacturers and one retailer compete simultaneously considering three factors including price, service and simple price discount contract. The preliminary results showed that the service and the price discount contract can improve the performance of supply chain. Chen et al. [24] investigated the interaction of provider of free after-sales service and contract type of either wholesale price contracts or consignment contracts with revenue sharing in a two-echelon supply chain with one manufacturer and one retailer facing random demand. Jena and Sarmah [25] studied price and service co-opetition among the two remanufacturing firms, which were competing on price and service to sell their substitutable products through a common retailer, and provide service directly to the end customers. Zhang and Wang [26] investigated two dynamic pricing strategies in a dual-channel supply chain consisting of a manufacturer, a retailer and focused on the influence of service value on the decisions and the insight of complexity. Ma et al. [27] examined the optimal decisions of dual-channel game model considering the inputs of retailing service, and analyzed how adjustment speed of service inputs affect the system complexity and market performance. Besides, Ghosh [28] discussed optimal pricing strategy of a two-echelon supply chain consisting of one manufacturer and two retailers with price and service sensitive demand.

The above literatures studied the impact of service level on the channel conflict and supply chain profits in dual-channel supply chain. But they seldom involved the research of the channel cooperation and service in the multi-channel supply chain. What will the existence of OBC have influence on the supply chain system? How does service level affect system stability and system effectiveness? So this paper will explore the influence of the channel cooperation and service on the multi-channel supply chain with OSC by using entropy theory, nonlinear dynamics theory and game theory. This paper builds two multi-channel dynamic price game models based on the Nash game model and Stackelberg game model respectively. We will study the equilibrium point, complexity entropy and efficiency of the system, and analysis the dynamic evolution of the system under the different system parameters by the numerical simulation.

In recent years, a few scholars have applied the theory of entropy to the research on economic and management [29,30], Lou et al. [31] analyzed the bullwhip effect in a supply chain with a sales game and consumer returns via the theory of entropy and complexity. Considering the current and the historical output, Han [32] focused on the influence of time delay parameter on the complexity of the system. Levner [33] presented the entropy-based optimization model for reducing the supply chain model size and assessing the economic loss. The main contributions of this paper are as follows:

(1) This paper builds a multi-channel supply chain model considering OSC and proposes a new perspective for multichannel research.
(2) This paper discusses the effect of service cooperation on the multi-channel supply chain with OSC and provides decision references for enterprises.
(3) This paper studies the complexity and characteristics of the multichannel service supply chain and puts forward management opinions.

This paper is organized as follows: Section 2 introduces the basic model description and symbolic representation. Section 3 presents and analyses the Nash dynamic game model. Section 4 presents and analyses the Stackelberg dynamic game model. The conclusions are given in Section 5.

## 2. Model Description 

### 2.1. Basic Model Description

**Figure 1:**
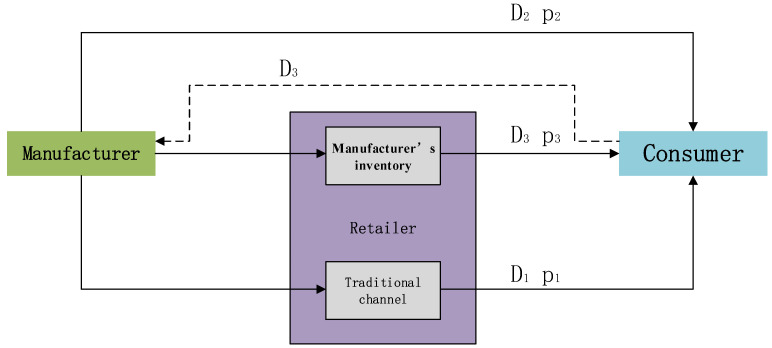
The multi-channel supply chain system.

Based on the channel cooperation and service, a new sales channel is considered in multi-channel supply chain with a dual-channel manufacturer and a traditional channel retailer (see Figure 1). The dual-channel manufacturer relies on three channels to provide same products for the market: the first is traditional channel where the retailer sells products to consumers at $p_{1}$ and provides service level $s_{1}$; the second is the online channel where the manufacturer sells products to consumers at $p_{2}$ and provides service level $s_{2}$; the third is OSC where the manufacturer sets a certain amount of stock at the retailer’s store, and allows the consumer to place an order in online channel at $p_{3}$ and then pick up at retailer’s store. In OSC, the retailer provides services for consumers, and the service level $s_{3}$ and product price $p_{3}$ are determined by the manufacturer. Assuming there is no moral hazard, the manufacturer and the retailer share the profit and cost of the OSC in proportion h which determines the profit of retailer from the OSC. When the value of h is too small, the retailer may not be willing to cooperate with manufacturer. Therefore, the manufacturer needs to negotiate with the retailer to determine the value of h.

### 2.2. Symbol Description

The following notations will be used in the paper:

$a_{i}$: the size of the potential market (i=1, 2, 3)
w: the wholesale price that the dual-channel manufacturer sets for the traditional retailer
b: the price sensitive coefficient
k: the influence of the substitute’s price
$D_{i}\left(t\right)$: product demand at period *t*(i=1, 2, 3)
$p_{i}\left(t\right)$: product price at period *t*(i=1, 2, 3)
$s_{i}$: the service level (i=1, 2, 3)
$\gamma_{i}$: the service sensitive coefficient (i=1, 2, 3)
*e*: influence of the substitute’s service
$\eta_{i}$: unit service cost (i=1, 2, 3)
$\pi_{m}\left(t\right)$: manufacturer’s profit at period *t*
  $\pi_{r}\left(t\right)$: retailer’s profit at period *t*
h: profit distribution rate

### 2.3. Profit Functions

The manufacturer and retailer both choose prices as the decision variables. Based on related literature [34,35,36] and the actual competition situation, the channel demand is affected by the product price and services of other channel. The demand functions in three channels are defined as follows:

(1) $$\left\{ \begin{array}{l} D_{1}\left(t\right)=a_{1}-bp_{1}\left(t\right)+kp_{2}\left(t\right)+kp_{3}\left(t\right)+\gamma_{1}s_{1}-es_{2}-es_{3}\\ D_{2}\left(t\right)=a_{2}-bp_{2}\left(t\right)+kp_{1}\left(t\right)+kp_{3}\left(t\right)+\gamma_{2}s_{2}-es_{1}-es_{3}\\ D_{3}\left(t\right)=a_{3}-bp_{3}\left(t\right)+kp_{2}\left(t\right)+kp_{1}\left(t\right)+\gamma_{3}s_{3}-es_{1}-es_{2} \end{array}\right.$$

Customers will decide which channel to buy products from according to the price and service level. A higher service level will bring about a larger cost. According to Tsay [37] and Li et al. [38], the cost of a service level is shown as follows:

(2) $$c\left(s_{i}\right)=\frac{\eta_{i}s_{i}^{2}}{2}$$

In order to simplify the model, assuming that the manufacturer’s production cost is zero, we can get the profit function of the three channels:

(3) $$\{\begin{array}{l} \pi_{1}\left(t\right)=\left(p_{1}\left(t\right)-w\right)D_{1}\left(t\right)-\frac{\eta_{1}s_{1}^{2}}{2}\\ \pi_{2}\left(t\right)=p_{2}\left(t\right)D_{2}\left(t\right)-\frac{\eta_{2}s_{2}^{2}}{2}\\ \pi_{3}\left(t\right)=p_{3}\left(t\right)D_{3}\left(t\right)-\frac{\eta_{3}s_{3}^{2}}{2} \end{array}$$

According to the contract, the manufacturer and retailer share the profit of the OSC in proportion h. Therefore, the profit functions of the manufacturer and retailer are as follows:

(4) $$\{\begin{array}{l} \pi_{r}\left(t\right)=\left(p_{1}\left(t\right)-w\right)D_{1}\left(t\right)-\frac{\eta_{1}s_{1}^{2}}{2}+h\pi_{3}\left(t\right)\\ \pi_{m}\left(t\right)=p_{2}\left(t\right)D_{2}\left(t\right)+wD_{1}\left(t\right)-\frac{\eta_{2}s_{2}^{2}}{2}+\left(1-h\right)\pi_{3}\left(t\right) \end{array}$$

## 3. The Nash Equilibrium Game Model

We give the Nash equilibrium game model in which the manufacturer and the retailer make decisions simultaneously and do not regard the reaction of the other.

### 3.1. Model Construction

We can get the marginal profits of the manufacturer and retailer as follows:

(5) $$\left\{ \begin{array}{l} \frac{\partial \pi_{r}\left(t\right)}{\partial p_{1}\left(t\right)}=a_{1}-2bp_{1}\left(t\right)+kp_{2}\left(t\right)+\left(1+h\right)kp_{3}\left(t\right)-es_{2}-es_{3}+\gamma_{1}s_{1}+bw\\ \frac{\partial \pi_{m}\left(t\right)}{\partial p_{2}\left(t\right)}=a_{2}-2bp_{2}\left(t\right)+kp_{1}\left(t\right)+\left(2-h\right)kp_{3}\left(t\right)+\gamma_{2}s_{2}-es_{1}-es_{3}+kw\\ \frac{\partial \pi_{m}\left(t\right)}{\partial p_{3}\left(t\right)}=kp_{2}\left(t\right)-\left(h-1\right)\left(a_{3}-es_{1}-es_{2}-2bp_{3}\left(t\right)+kp_{1}\left(t\right)+kp_{2}\left(t\right)+\gamma_{3}s_{3}\right)+kw \end{array}\right.$$

The best reply functions of the manufacturer and retailer are as follows:

$$p_{1}^{*}=\frac{-\left(M_{1}C_{1}+M_{2}C_{2}+M_{3}C_{3}\right)}{M}$$

$$p_{2}^{*}=\frac{-\left(T_{1}C_{1}+T_{2}C_{2}+T_{3}C_{3}\right)}{M}$$

$$p_{3}^{*}=\frac{-\left(U_{1}C_{1}+U_{2}C_{2}+U_{3}C_{3}\right)}{M}$$

where:

$M=8b^{3}h+12bk^{2}-2hk^{3}-8b^{3}+4k^{3}-10bhk^{2}$;
$M_{1}=4b^{2}-4k^{2}-k^{2}h^{2}-4b^{2}h+4hk^{2}$;
$M_{2}=2k^{2}-h^{2}k^{2}+2bk+hk^{2}-2bhk$;
$M_{3}=2k^{2}+2bk-hk^{2}+2bhk$;
$T_{1}=2k^{2}+h^{2}k^{2}+2bk-3hk^{2}$;
$T_{2}=4b^{2}-k^{2}+h^{2}k^{2}-4b^{2}h$;
$T_{3}=k^{2}+4bk+hk^{2}-2bhk$;
$U_{1}=2k^{2}+2bk-hk^{2}-2bhk$;
$U_{2}=k^{2}+4bk-hk^{2}-2bhk$;
$U_{3}=4b^{2}-k^{2}$.

The market information has more commercial value in a real market, but it is impossible for the manufacturer and retailer to get all the market information, so the manufacturer and retailer have limited rational behavior when making decisions, and they adjust decision variable $p_{i}$ to reach a dynamic equilibrium state based on the marginal profit in the previous period. When the manufacturer and retailer realize that the marginal profit in period t is positive, they will increase the price in period t+1 to gain more profit; when the marginal profit is negative, they will decrease the price in period t+1. The adjustment rule is called bounded rationality expectation rule (BRE).

The BRE rule is widely used to describe the dynamic decision process in the economic system [39,40]. Assuming that the manufacturer and retailer are both bounded rational, they make decisions of the price in the next period on the basis of their marginal profits. Then, the dynamic game process of the manufacturer and retailer can be modeled as the following dynamic nonlinear system:

(6) $$\left\{ \begin{array}{l} p_{1}\left(t+1\right)=p_{1}\left(t\right)+\alpha_{1}p_{1}\left(t\right)\left[C_{1}-2bp_{1}\left(t\right)+kp_{2}\left(t\right)+\left(1+h\right)kp_{3}\left(t\right)\right]\\ p_{2}\left(t+1\right)=p_{2}\left(t\right)+\alpha_{2}p_{2}\left(t\right)\left[C_{2}-2b_{2}p_{2}\left(t\right)+kp_{1}\left(t\right)+\left(2-h\right)kp_{3}\left(t\right)\right]\\ p_{3}\left(t+1\right)=p_{3}\left(t\right)+\alpha_{3}p_{3}\left(t\right)\left\{C_{3}+kp_{2}\left(t\right)-\left(h-1\right)\left[-2b_{3}p_{3}\left(t\right)+kp_{1}\left(t\right)+kp_{2}\left(t\right)\right]\right\} \end{array}\right.$$

where:

$$C_{1}=a_{1}-es_{2}-es_{3}+\gamma_{1}s_{1}+bw$$

$$C_{2}=a_{2}-es_{1}-es_{3}+\gamma_{2}s_{2}+kw$$

$$C_{3}=\left(1-h\right)\left(a_{3}-es_{1}-es_{2}+\gamma_{3}s_{3}\right)+kw$$

where $\alpha_{i},\;i=1,\;2,\;3$ is the adjustment speed parameters of prices of the manufacturer and retailer, which reflects the company agents’ learning and active managerial behavior.

### 3.2. The Stability of the System (6)

#### 3.2.1. System Equilibrium Points

The different of the system (6) with the linear system is that the nonlinear system (6) has multiple equilibriums. By setting $p_{i}\left(t+1\right)=p_{i}\left(t\right)$, the eight equilibrium points of the system (6) can be obtained:

$E_{1}=\left(0,\;0,\;0\right)$
$E_{2}=\left(\frac{C_{1}}{2b},\;0,\;0\right)$
$E_{3}=\left(0,\;0,\;\frac{C_{3}}{2b-2bh}\right)$
$E_{4}=\left(0,\frac{C_{2}}{2b},0\right)$
$E_{5}=\left(\frac{2C_{1}b+C_{2}k}{4b^{2}-k^{2}},\frac{2C_{2}b_{1}+C_{1}k}{4b^{2}-k^{2}},0\right)$
$E_{6}=\left(\frac{2C_{1}bh-C_{3}hk-2C_{1}b-C_{3}k}{4b^{2}h-h^{2}k^{2}-4b^{2}+k^{2}},0,\frac{-2C_{3}b-C_{1}k+C_{1}hk}{4b^{2}h-h^{2}k^{2}-4b^{2}+k^{2}}\right)$
$E_{7}=\left(0,\frac{-2C_{2}b\left(1-h\right)+C_{3}k\left(2-h\right)}{k^{2}\left(4+h^{2}-4h\right)+4b^{2}\left(h-1\right)},\frac{-2C_{3}b+C_{2}k\left(h-2\right)}{k^{2}\left(4+h^{2}-4h\right)+4b_{2}b_{3}\left(h-1\right)}\right)$
$E^{*}=\left(p_{1}^{*},p_{2}^{*},p_{3}^{*}\right)$

#### 3.2.2. Stability Analysis of the Equilibrium Points

As the boundary equilibrium points ($p_{i}=0$) are meaningless, we guess that $E_{1}$, $E_{2}$, $E_{3}$, $E_{4}$, $E_{5}$, $E_{6}$, $E_{7}$ are boundary equilibrium points, $E^{*}$ is the only Nash equilibrium point. The proof is shown as follows:

The Jacobi matrix of the system (6) is:

(7) $$J=\left[\begin{array}{ccc} J_{11} & k\alpha_{1}p_{1}\left(t\right) & k\alpha_{1}\left(1+h\right)p_{1}\left(t\right)\\ k\alpha_{2}p_{2}\left(t\right) & J_{22} & k\alpha_{2}\left(2-h\right)p_{2}\left(t\right)\\ -k\alpha_{3}\left(h-1\right)p_{3}\left(t\right) & k\alpha_{3}p_{3}-k\alpha_{3}\left(h-1\right)p_{3}\left(t\right) & J_{33} \end{array}\right]$$

where:

$$J_{11}=1+\alpha_{1}\left[C_{1}-2bp_{1}\left(t\right)+kp_{2}\left(t\right)+k\left(1+h\right)p_{3}\left(t\right)\right]$$

$$J_{22}=1+\alpha_{2}\left[C_{2}+kp_{1}\left(t\right)-2bp_{2}\left(t\right)+k\left(2-h\right)p_{3}\left(t\right)\right]$$

$$J_{33}=1+\alpha_{3}\left\{C_{3}+kp_{2}\left(t\right)-\left(h-1\right)\left[-4bp_{3}\left(t\right)+kp_{1}\left(t\right)+kp_{2}\left(t\right)\right]\right\}$$

The characteristic values at $E_{1}$ are:

$$\lambda_{1}=1+\alpha_{1}C_{1}$$

$$\lambda_{2}=1+\alpha_{2}C_{2}$$

$$\lambda_{3}=1+\alpha_{3}C_{3}$$

Because $D_{1}\left(t\right)>0$, $D_{2}\left(t\right)>0$, $D_{3}\left(t\right)>0$, we sort out the demand function and get the following expression:

$$C_{1}=a_{1}-es_{2}-es_{3}+\gamma_{1}s_{1}+bw>bp_{1}-kp_{2}-kp_{3}+bw=bw>0$$

$$C_{2}=a_{2}-es_{1}-es_{3}+\gamma_{2}s_{2}+kw>bp_{2}-kp_{1}-kp_{3}+kw=kw>0$$

$$C_{3}=-\left(h-1\right)\left(a_{3}-es_{1}-es_{2}+\gamma_{3}s_{3}\right)+kw>-\left(h-1\right)\left(bp_{3}-kp_{1}-kp_{2}\right)+kw=kw>0$$

It is easily concluded that $\lambda_{1}>1,\;\lambda_{2}>1,\;\lambda_{3}>1$. $E_{1}$ is an unstable saddle point. Similarly, $E_{i}\left(i=2,\;3,\;4,\;5,\;6,\;7\right)$ can be proved to be unstable saddle points, $E^{*}$ is the only Nash equilibrium point.

At the Nash equilibrium point $E^{*}$, the Jacobi matrix is as follows:

$$J\left(E^{*}\right)=\left[\begin{array}{ccc} J_{11}^{*} & k\alpha_{1}p_{1}^{*}\left(t\right) & k\alpha_{1}\left(1+h\right)p_{1}^{*}\left(t\right)\\ k\alpha_{2}p_{2}^{*}\left(t\right) & J_{22}^{*} & k\alpha_{2}\left(2-h\right)p_{2}^{*}\left(t\right)\\ -k\alpha_{3}\left(h-1\right)p_{3}^{*}\left(t\right) & k\alpha_{3}p_{3}-k\alpha_{3}\left(h-1\right)p_{3}^{*}\left(t\right) & J_{33}^{*} \end{array}\right]$$

The characteristic equation of $J\left(E^{*}\right)$ is:

(8) $$f\left(\lambda \right)=\lambda^{3}+A\lambda^{2}+B\lambda +C$$

where:

$$A=-J_{11}^{*}-J_{22}^{*}-J_{33}^{*}$$

$$B=J_{33}^{*}J_{11}^{*}+J_{33}^{*}J_{22}^{*}+J_{11}^{*}J_{22}^{*}-J_{12}^{*}J_{21}^{*}-J_{13}^{*}J_{31}^{*}-J_{23}^{*}J_{32}^{*}$$

$$C=J_{13}^{*}J_{31}^{*}J_{22}^{*}+J_{23}^{*}J_{32}^{*}J_{11}^{*}-J_{31}^{*}J_{12}^{*}J_{23}^{*}-J_{33}^{*}J_{11}^{*}J_{22}^{*}+J_{33}^{*}J_{12}^{*}J_{21}^{*}-J_{32}^{*}J_{13}^{*}J_{21}^{*}$$

According to the July criterion, the necessary and sufficient condition for the locally stability of $E^{*}$ is as follows:

(9) $$\{\begin{array}{l} f\left(1\right)>0\\ \begin{array}{l} {\left(-1\right)}^{3}f\left(-1\right)>0\\ \left|A\right|<1 \end{array}\\ \left|CA-B\right|<\left|C^{2}-1\right| \end{array}$$

By solving condition (9), the stability domain of the system (6) can be obtained. Due to these limitations being so complex, solving the inequality equation (9) is very complicated. If the Nash equilibrium point satisfies the inequality equation (9), we may ensure that the system (6) is locally stable. Next, we give the stable characteristics of the system (6) through numerical simulation.

### 3.3. Numerical Simulation

This paper investigates the dynamic characteristics of the system (6) using the numerical simulation method, such as the stable region, bifurcation, chaos, and chaotic attractor. According to the actual competition, we take the basic parameter values as follows: $a_{1}=50,\;a_{2}=55,\;a_{3}=18,\;b=1.3,\;\eta_{1}=0.5,\;\eta_{2}=0.8,\;\eta_{3}=0.7,\;\gamma_{1}=0.6,\;\gamma_{2}=0.4,\;\gamma_{3}=0.5,\;s_{1}=2,\;s_{2}=5,\;s_{3}=3,\;h=0.6,\;w=17,\;k=0.4,\;e=0.1$. We can get $E^{*}=\left(44.503,40.307,42.320\right)$.

#### 3.3.1. Stability Region of the System (6)

**Figure 2:**
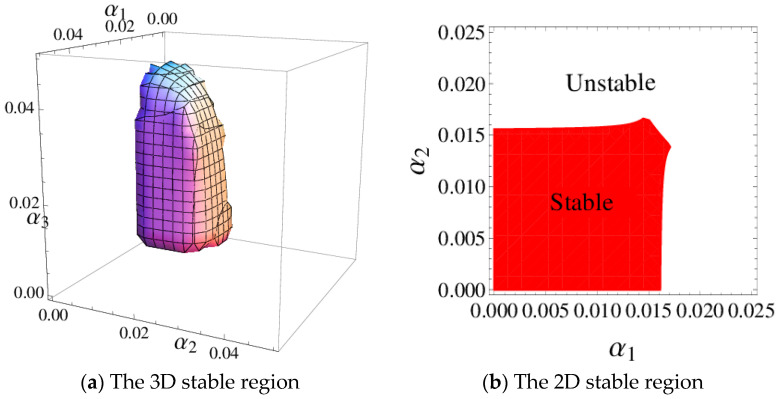
The stable region of the system (6).

According to the Jury stability criterion which is given in inequality (9), Figure 2a shows the 3D stability region of the system (6); It means that the system (6) will converge to the point $E^{*}$ after the finite evolutionary when $\alpha_{1},\alpha_{2}\;\mathrm{and}\;\alpha_{3}$ take values in the 3D stable region unless some factors outside break the equilibrium state. Figure 2b depicts the 2D stable region of system (6) when $\alpha_{3}=0.03$, the system (6) will converge to the point $E^{*}$ after the finite evolutionary when $\alpha_{1}\;\mathrm{and}\;\alpha_{2}$ take values in the 2D stable region when $\alpha_{3}=0.03$.

**Figure 3:**
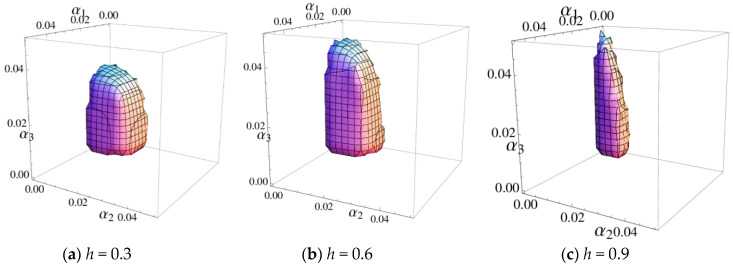
The 3D stable region of the system (6).

**Figure 4:**
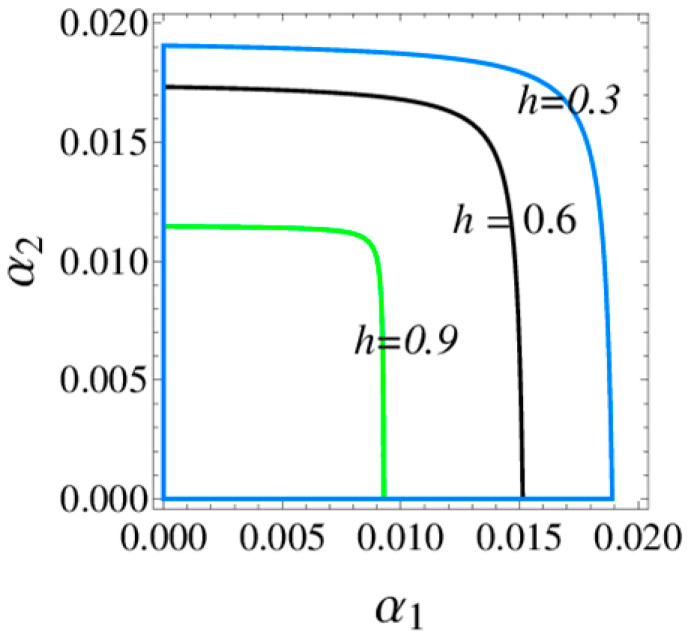
The stable region of the system (6) when $\alpha_{3}=0.02$.

Figure 3 shows the 3D stable regions of the system (6) with the different values of h and Figure 4 shows 2D stable regions when $\alpha_{3}=0.02$, it clearly indicates that the stable ranges of $\alpha_{1}\;\mathrm{and}\;\alpha_{2}$ decrease and the stable range of $\alpha_{3}$ increases with h increasing, so the retailer should choose an appropriate distribution rate to make the system (6) be in a stable state. 

#### 3.3.2. The Influence of the Price Adjustment Speed on the System Stability

**Figure 5:**
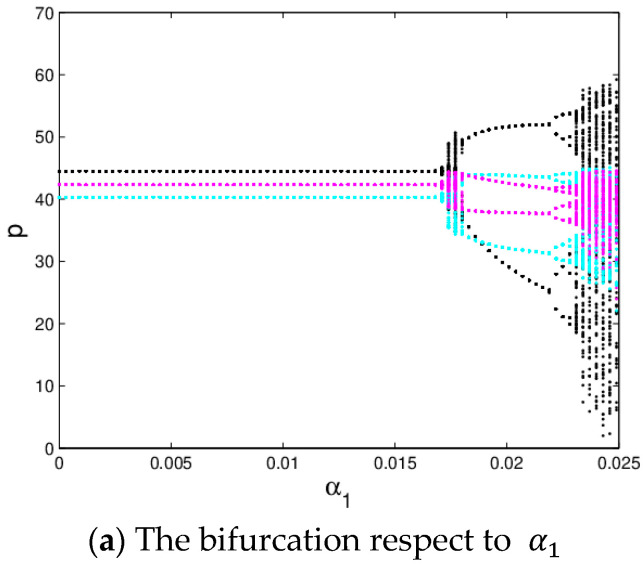

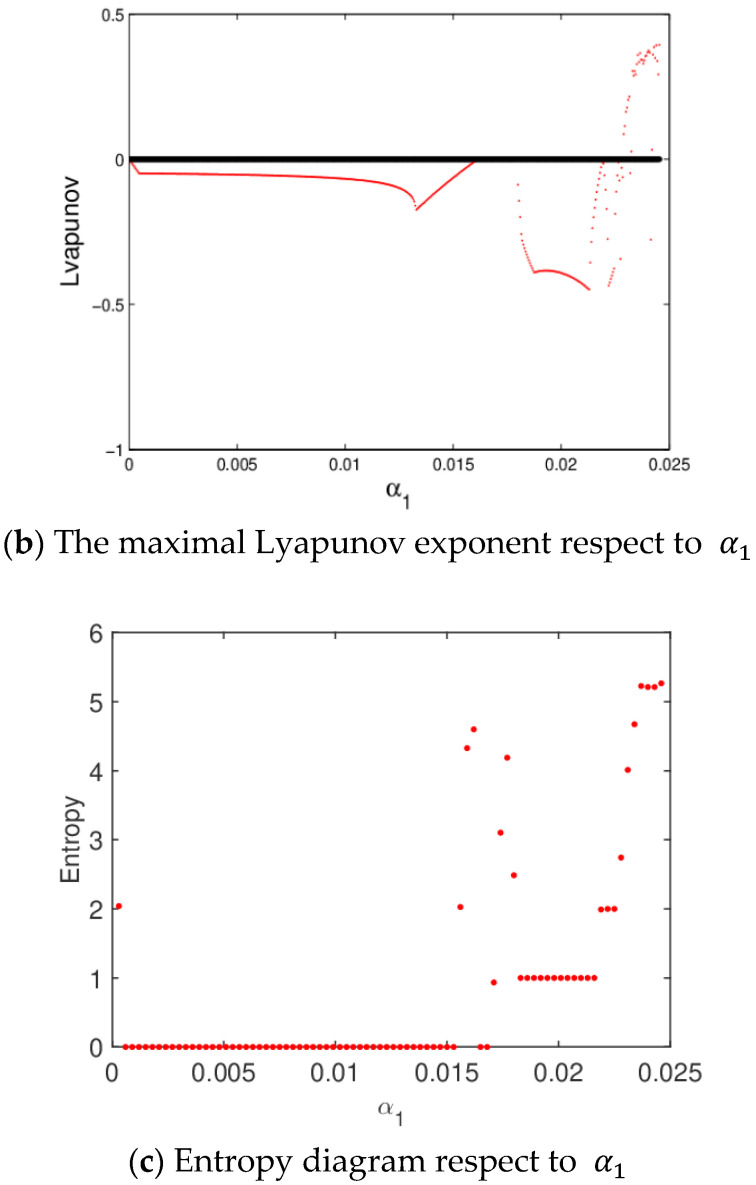
The evolution process of the system (6).

Figure 5a shows the bifurcation diagram of the system (6) when $\alpha_{2}=0.015,\alpha_{3}=0.03$ with $\alpha_{1}$ varying from 0 to 0.025. The black line is the evolution process of $p_{1}$, the blue green line represents the evolution process of $p_{2}$ and the red line represents the evolution process of $p_{3}$. As shown in Figure 5, when $\alpha_{1}<0.0175$, the system (6) is stable; when $\alpha_{1}>0.0175$, the system (6) falls into chaos experiencing the 4-period bifurcation, 4-period bifurcation, 8-period bifurcation, etc. Figure 5b gives the corresponding LLE of the system (6), the LLE is less than zero when $\alpha_{1}$ < 0.0226; with the growth of $\alpha_{1}$, the LLE becomes positive which means the system (6) enters into chaotic state, it is consistent with Figure 5a. Figure 5c is the entropy diagram of the system (6), we can find that the system entropy is equal to zero when the system (6) is in the quasi-stable state ($\alpha_{1}$ < 0.0175), and the LLE is less than zero simultaneously. Once the system (6) enters into flip bifurcation stage ($\alpha_{1}$ > 0.0175), the system entropy will increase. The more chaotic the system (6) is, the greater the entropy of the system (6) is.

**Figure 6:**
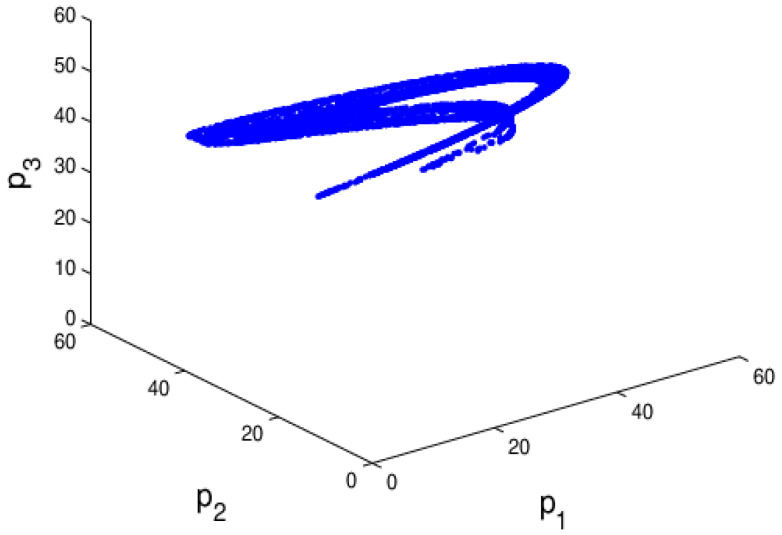
The chaotic attractor of the system (6) when $\alpha_{1}$ = 0.024, $\alpha_{2}=0.015$, $\alpha_{3}=0.03$.

**Figure 7:**
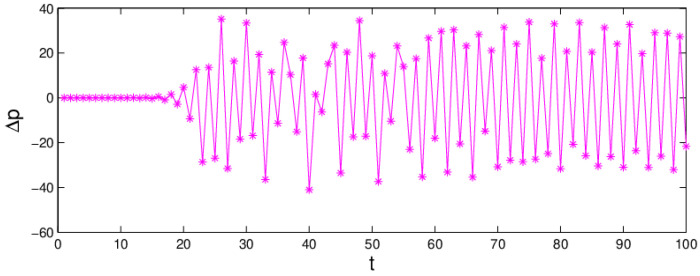
The sensitivity to initial values of the system (6) when $\alpha_{1}$ = 0.024, $\alpha_{2}=0.015$, $\alpha_{3}=0.03$.

The chaotic attractor and the sensitivity to the initial value are important characteristics when a system is in chaos. If the attractor is a fixed point, the system is in a stable state. On the contrary, when the attractor is not a fixed point and period cycles, the system is in chaos. From the bifurcation diagram, the largest Lyapunov exponent, and system entropy, we may see that the system (6) is in chaotic state when $\alpha_{1}$ = 0.024, $\alpha_{2}=0.015$ and $\alpha_{3}=0.03$ and the chaotic attractor is given in Figure 6. In chaotic state, the system (6) becomes unstable and complex. Correspondingly, Figure 7 shows the sensitivity to the initial value with a little change in $p_{1}$. When $p_{1}$ only changes 0.0001, the system (6) will show a significant difference after about 17 iteration cycles. So, when the system is in chaotic state with high entropy, a minor change in the initial value will cause a huge difference in later decisions.

By analyzing the influence of the price adjustment speed on the stability of the system, we can conclude that the manufacturer and the retailer should not take an overlarge price adjustment speed when making price decisions; otherwise the system will fall into an unstable state with high entropy, which is harmful to the correct decisions of the manufacturer and retailer. Because the change of $\alpha_{2}$ and $\alpha_{3}$ has the same effect on the system with the change of $\alpha_{1}$, no further description is given here.

#### 3.3.3. The Influence of Service Level on the System Stability 

**Figure 8:**
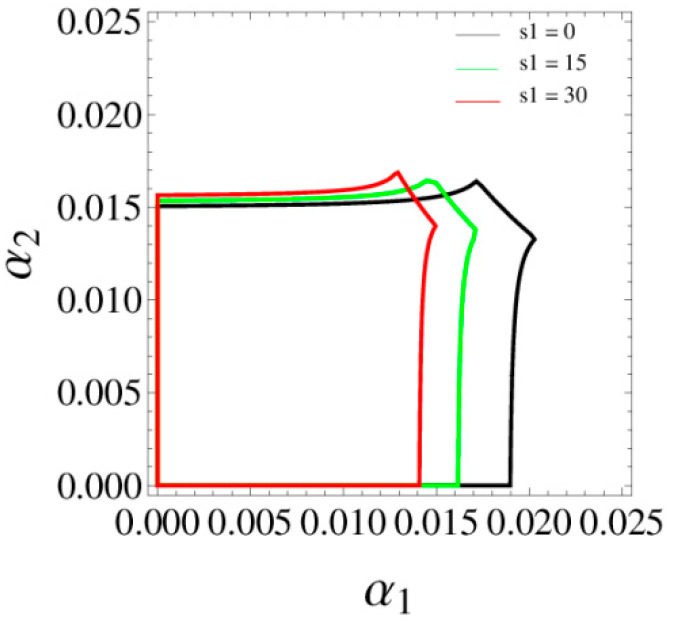
The stable region respect to $\alpha_{1}$ and $\alpha_{2}$ when $s_{1}=0,\;15,\;30$.

**Figure 9:**
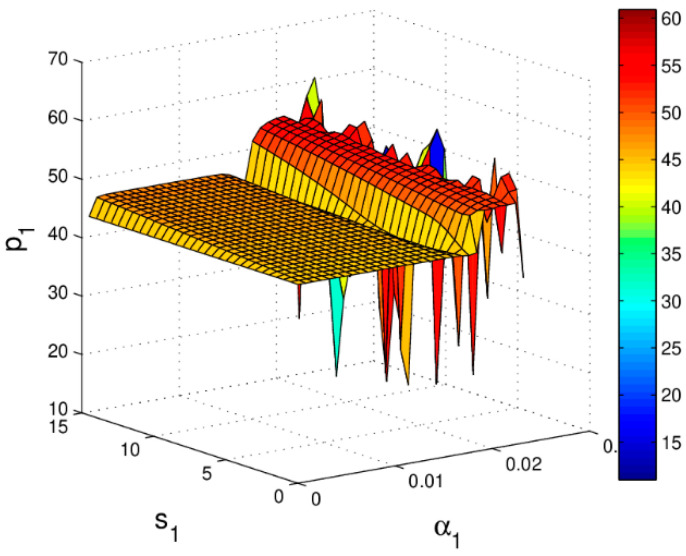
The change of $p_{1}$ with respect to $\alpha_{1}\;\mathrm{and}\;s_{1}$.

Figure 8 shows the stable regions of $\alpha_{1}\;\mathrm{and}\;\alpha_{2}$ with change of $s_{1}$, we can see that the stable range of $\alpha_{1}$ decreases and there is almost no change in the stability range of $\alpha_{2}$ with the increase of $s_{1}$. Figure 9 gives the 3D price evolution with the change of $s_{1}\;\mathrm{and}\;\alpha_{1}$. The first bifurcation occurs at $\alpha_{1}=0.0162$ when $s_{1}=0$; the first bifurcation occurs at $\alpha_{1}=0.0158$ when $s_{1}=15$. We can see clearly that the higher the service level is, the easier the system bifurcation occurs. What’s more, it can easily find that $p_{1}$ increases from 43.94 to 48.79 when $s_{1}$ changes from 0 to 15.

Above all, we can get that the service level has obvious impact on the dynamic stability characteristics of the system (6). The high service level decreases the stability domain of the system (6) and causes the system (6) falls into chaos earlier. Because the changes of $s_{2}$ and $s_{3}$ have the same effect on the system with the change of $s_{1}$, no further description is given here.

#### 3.3.4. The Influence of the Service Level on System Profit

**Figure 10:**
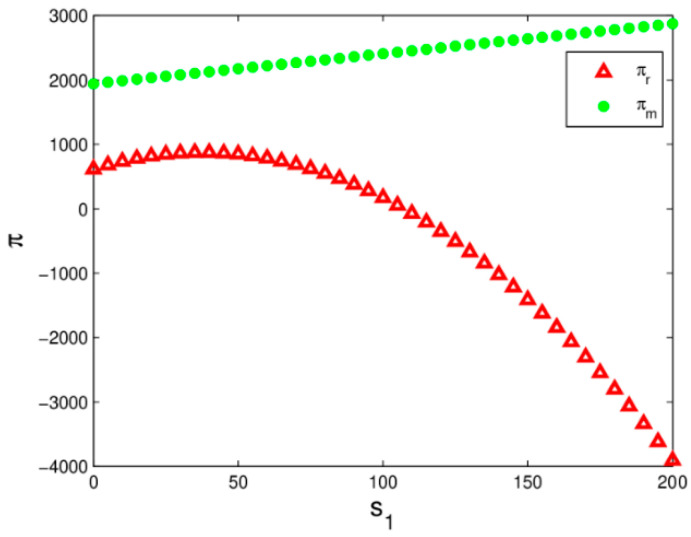
The profit evolution respect to $s_{1}$ when the system (6) is in stable state.

Figure 10 shows the profit changes of the manufacturer and retailer with $s_{1}$ increasing when the system (6) is in a stable state. The manufacturer’s profit increases and the retailer’s profit increases first and then decreases with $s_{1}$ increasing in which the main reason is that the increase of $s_{1}$ has caused a raise in demand for the manufacturer and retailer, but the retailer’s cost increase with $s_{1}$ increases. The retailer should choose the best service level to maximize his profit.

**Figure 11:**
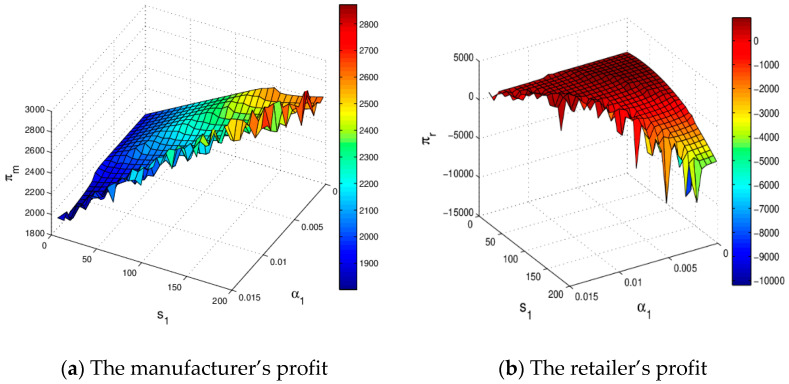
The change of profit with respect to $\alpha_{1}\;\mathrm{and}\;s_{1}$ when $\alpha_{2}=0.015$, $\alpha_{3}=0.03$.

Figure 11 shows the impact of $s_{1}$ and $\alpha_{1}$ on profits of the manufacturer and retailer. When $s_{1}$ is relatively high, with the increase of $\alpha_{1}$, the profits of the manufacturer and retailer will be more easily destroyed. When $\alpha_{1}$ and $s_{1}$ are maintained at an appropriate range, the profits of the manufacturer and retailer are stable. It can be clearly seen that the efficiency of the system (6) in the chaotic state is obviously lower than that in the steady state system.

**Figure 12:**
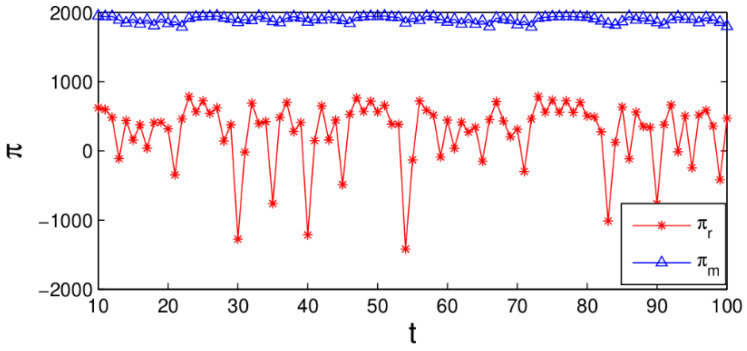
The time series of profit when $\alpha_{1}=0.024,\alpha_{2}=0.015,\alpha_{3}=0.03$.

Figure 12 shows the change of profit over time when the system (6) is in a chaotic state, the retailer’s profit has obvious fluctuation which illustrates that chaos destroys the market order and reduces system’s efficiency. We can conclude that the service input plays an important role in gaining profit. In the market competition, the manufacturer and retailer keeping the values of parameters in the appropriate range is conducive to the stability and improvement of the system’s profit.

## 4. The Stackelberg Dynamic Game Model

### 4.1. The Model Construction

In this section, supposing that the manufacturer and the retailer have principal and subordinate relationship considering the channel service, the manufacturer is Stackelberg leader and the retailer is follower. Then, the manufacturer and retailer process sequential dynamic game; the game equilibrium is the Stackelberg equilibrium. In this game, the manufacturer makes price decisions $p_{2}$ and $p_{3}$ for the direct channel and the “online-store” channel according to the market information; the retailer makes decisions for $p_{1}$ according to the manufacturer’ decisions. Customers will decide which channel to buy products according to the price and service level. So we can get the profit function of manufacturer and retailer as follows:

Using backward induction, we first find the response function of the retailer in the second stage from the game model, which can be obtained by the first order conditions of Equation (2); the equilibrium price of the retailer is as follows:

(10) $$p_{1}^{*}\left(t\right)=\frac{C_{1}+kp_{2}\left(t\right)+\left(1+h\right)kp_{3}\left(t\right)}{2b}$$

Equation (10) is the optimal decision making of the retailer on the premise of $p_{2}$ and $p_{3}$; the retailer can obtain the decision after it observes the manufacturer’s behavior.

Substituting Equation (10) into $\pi_{m}$ in Equation (4), the optimal price of the manufacturer can be obtained by the first order conditions of $\pi_{m}^{*}$ for $p_{2}$ and $p_{3}$. The optimal prices express optimal decisions of the manufacturer and retailer in various possible situations in a game stage.

In the actual decision process, the manufacturer and retailer show limited rational characteristics because they cannot obtain the perfect market information, so they make decisions for the next period based on the marginal profits of this period. The dynamic game process of the manufacturer and retailer can be modeled as the following dynamic system:

(11) $$\left\{ \begin{array}{l} p_{1}\left(t+1\right)=p_{1}\left(t\right)+\beta_{1}p_{1}\left(t\right)\frac{\partial \pi_{r}\left[p_{1}\left(t\right),\;p_{2}\left(t\right),\;p_{3}\left(t\right),\right]}{\partial p_{1}\left(t\right)}\\ p_{2}\left(t+1\right)=p_{2}\left(t\right)+\beta_{2}p_{2}\left(t\right)\frac{\partial \pi_{m}^{*}\left[p_{1}\left(t\right),\;p_{2}\left(t\right),p_{3}\left(t\right),\;\right]}{\partial p_{2}\left(t\right)}\\ p_{3}\left(t+1\right)=p_{3}\left(t\right)+\beta_{3}p_{3}\left(t\right)\frac{\partial \pi_{m}^{*}\left[p_{1}\left(t\right),\;p_{2}\left(t\right),\;p_{3}\left(t\right),\right]}{\partial p_{3}\left(t\right)} \end{array}\right.$$

where $\beta_{i}>0,\;i=1,\;2,\;3$ is the adjustment coefficients of $p_{1}$, $p_{2}$ and $p_{3}$. According to the dynamic adjustment process, we can see that the prices are related to the price adjustment speed, the prices of the competitor and channel service.

### 4.2. Equilibrium Points

By setting $p_{i}\left(t+1\right)=p_{i}\left(t\right)$, we can get the eight equilibrium points of the dynamic system (11). As the boundary equilibrium points are meaningless, we only consider the Nash equilibrium point:

$$P^{*}=\left(\frac{-\left(C_{1}H_{1}+C_{2}H_{2}+C_{3}H_{3}\right)}{Q},\frac{-\left(C_{2}L_{1}+C_{3}L_{2}\right)}{Q},\frac{-\left(C_{2}G_{1}+C_{3}G_{2}\right)}{Q}\right)$$

where:

$H_{1}=4b^{4}-8b^{2}k^{2}-h^{2}k^{4}-4hb^{4}-4bk^{3}+2bhk^{3}+6b^{2}hk^{2}+b^{2}h^{2}k^{2}$;
$H_{2}=2b^{2}k^{2}+2b^{3}k+bhk^{3}-2b^{3}hk+b^{2}hk^{2}+bh^{2}k^{3}-b^{2}h^{2}k^{2}$;
$H_{3}=2b^{2}k^{2}+2b^{3}k-bhk^{3}+2b^{3}hk-b^{2}hk^{2}$;
$L_{1}=2b^{3}-bk^{2}+bh^{2}k^{2}-2b^{3}h$;
$L_{2}=bk^{2}+2bk-b^{2}hk$;
$G_{1}=bk^{2}+2bk-b^{2}hk$;
$G_{2}=2b^{3}-bk^{2}$;
$C_{2}=a_{2}-es_{1}-es_{3}+\gamma_{2}s_{2}+\frac{kw}{2}+\frac{kC_{1}}{2b}$;
$C_{3}=wk-\left(h-1\right)\left(a_{3}-es_{1}-es_{2}+\gamma_{3}s_{3}+\frac{kC_{1}}{2b}\right)-\frac{wk\left(h+1\right)}{2}$;
$Q=4b^{4}h-4b^{4}-b^{2}h^{2}k^{2}-6b^{2}hk^{2}+8b^{2}k^{2}-2bhk^{3}+4bk^{3}+h^{2}k^{4}$.

Next, we will analyze the stable characteristic of the equilibrium point by the Jury stability criterion and study the influences of parameters values on stability of the system (11) by numerical simulation.

### 4.3. Numerical Simulation

The main goal of this section is to study the evolution characteristics of the system (11) under different settings, and then provides some management insights for firms. We also set values of parameters the same as in the system (6) for comparison, the equilibrium value of the system (11) is $P^{*}=\left(44.5963,\;40.4390,\;42.6167\right)$.

#### 4.3.1. Stability Region of the System (11)

**Figure 13:**
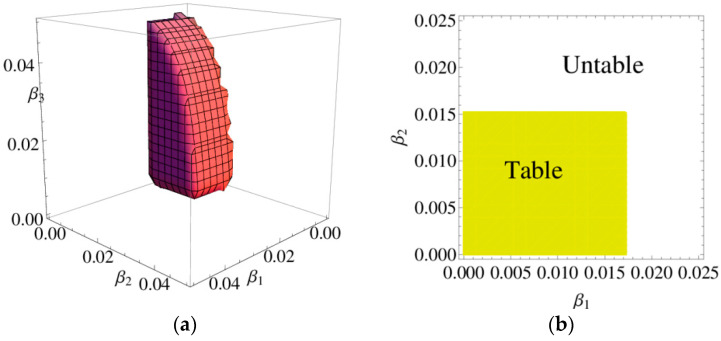
The stability region of the system at the Nash equilibrium point.

Figure 13a shows the stability region of the system (11), in which the system (11) will converge to the equilibrium point after the long-term evolutionary when $\beta_{1},\beta_{2}\;\mathrm{and}\;\beta_{3}$ take values in the 3D stable region unless some factors outside break the equilibrium state. Figure 13b depicts the stable region of the system (3) with yellow range and the unstable of the system with white region when $\beta_{3}=0.03$, we can clearly see what values of $\beta_{1}$ and $\beta_{2}$ can make the system (11) be in a stable state.

#### 4.3.2. The Influence of the Price Adjustment Speed on the System Stability

**Figure 14:**
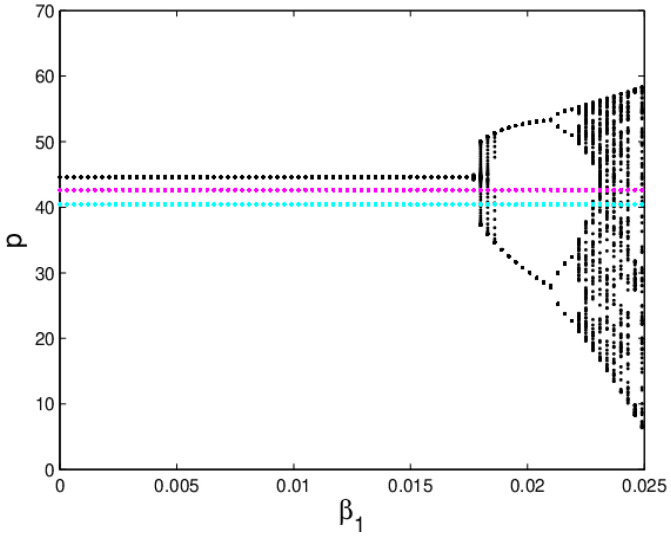
The bifurcation respect to $\beta_{1}$ when $\alpha_{2}=0.015$, $\alpha_{3}=0.03$.

**Figure 15:**
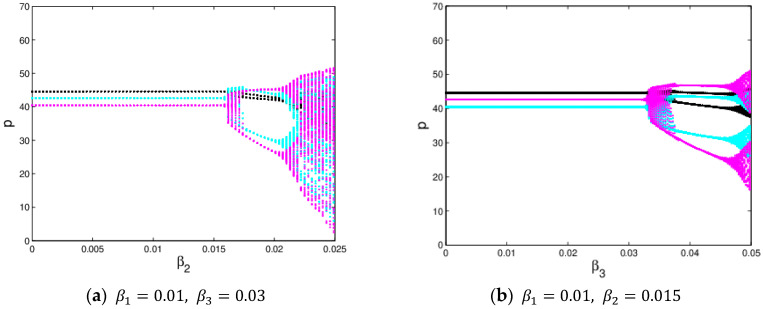
The evolution process of the system (11) with the change of $\beta_{2}\;\mathrm{and}\;\beta_{3}$

Figure 14 shows the price evolution process of the system (11) with $\beta_{1}$ varying from 0 to 0.025 when $\beta_{2}=0.015\;\mathrm{and}\;\beta_{3}=0.03$. The black line is the evolution process of $p_{1}$, the blue green line represents the evolution process of $p_{2}$ and the purple red line represents the evolution process of $p_{3}$. As shown in Figure 14, when $\beta_{1}\leq 0.0175$, the sales prices of the manufacturer and retailer are stable and the system entropy is low. Once $\beta_{1}>0.0175$, $p_{1}$ is unstable, $p_{2}$ and $p_{3}$ are also kept in stable state, which illustrates that the price adjustment of the retailer has little influence on the price evolution of the manufacturer. Figure 15 shows the price evolution process of the system (11) with $\beta_{2}$ and $\beta_{3}$ changing. We can see that the prices of the manufacturer and retailer finally falls into chaos through flip bifurcation, which illustrates that the price adjustment of the manufacturer will have an influence on the price evolution of the retailer.

**Figure 16:**
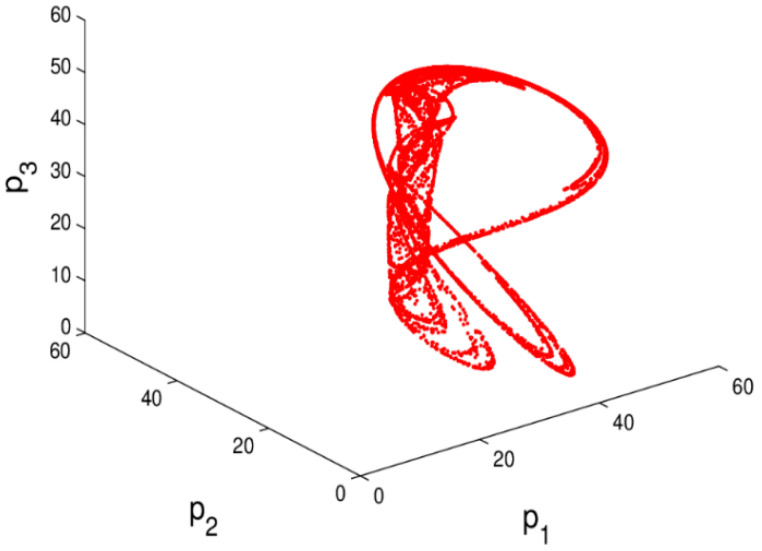
The attractor of the system (11) when $\beta_{1}=0.01$, $\beta_{2}$ = 0.024, $\beta_{3}=0.03$.

**Figure 17:**
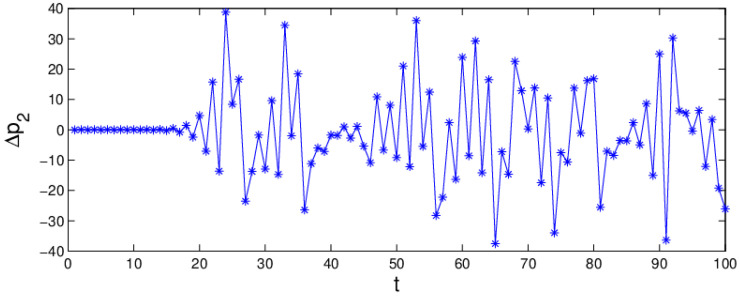
Sensitivity dependence to initial conditions with $p_{2}=40.4390$ and $p_{2}=40.4391$.

The chaotic attractor and sensitivity dependence to initial conditions investigate the chaotic characteristics of the system. If the attractor is a fixed point, the system is in stable state. On the contrary, when the attractor is not a fixed point and period cycle, the system is in chaos. Through the above analysis, the system (11) is in chaotic state when $\beta_{1}$ = 0.001, $\beta_{2}=0.024$ and $\beta_{3}=0.03$, its chaotic attractor is given in Figure 16; the system (11) becomes complex and the dynamic characteristics of the system (11) are more obviously. Correspondingly, Figure 17 shows the sensitivity dependence to initial conditions, the system (11) shows a significant difference when $p_{2}$ only changes 0.0001. So when the system is in chaos, even a minor change in the initial value will cause a huge difference in later decisions.

In short, the price adjustment speed of the retailer has great effect on its price evolution while little effect on the one of the manufacturer; the price adjustment speed of the manufacturer will have a huge influence on the price evolution of the retailer. The manufacturer and the retailer should not take an overlarge price adjustment speed when making price decision; otherwise the system will fall into an unstable state with high entropy.

#### 4.3.3. The Influence of Service Level on the System Stability

**Figure 18:**
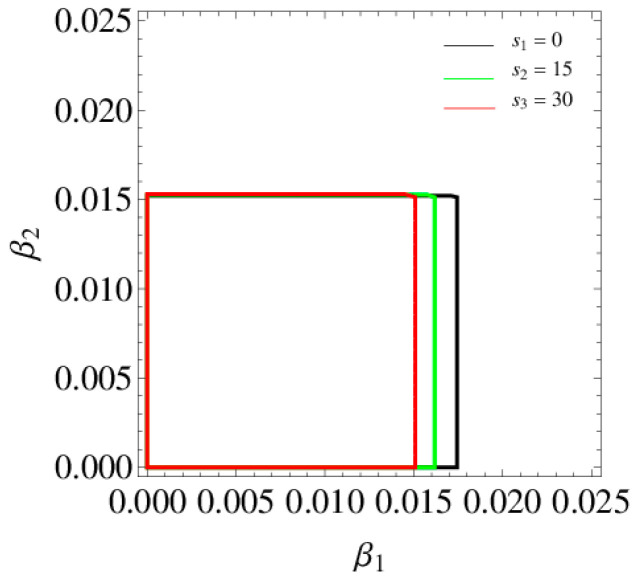
The stable regions respect to $\beta_{1}$ and $\beta_{2}$ when $s_{1}=0,\;15\;\mathrm{and}\;30$.

**Figure 19:**
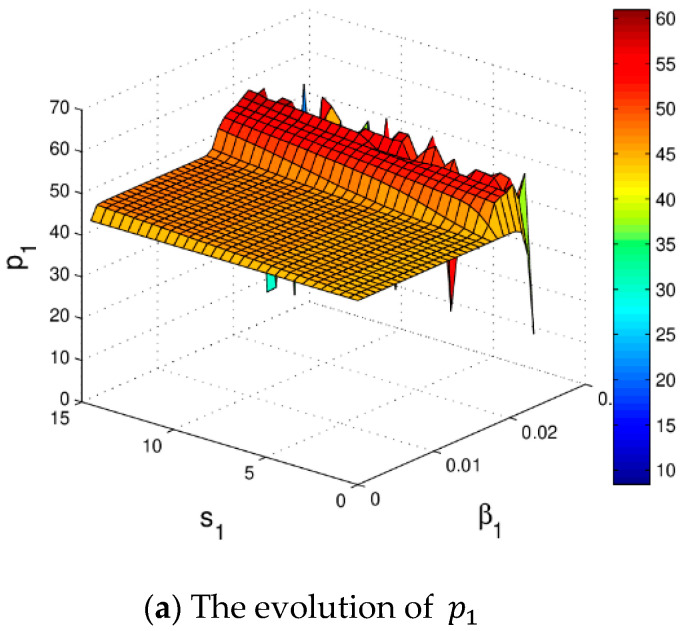

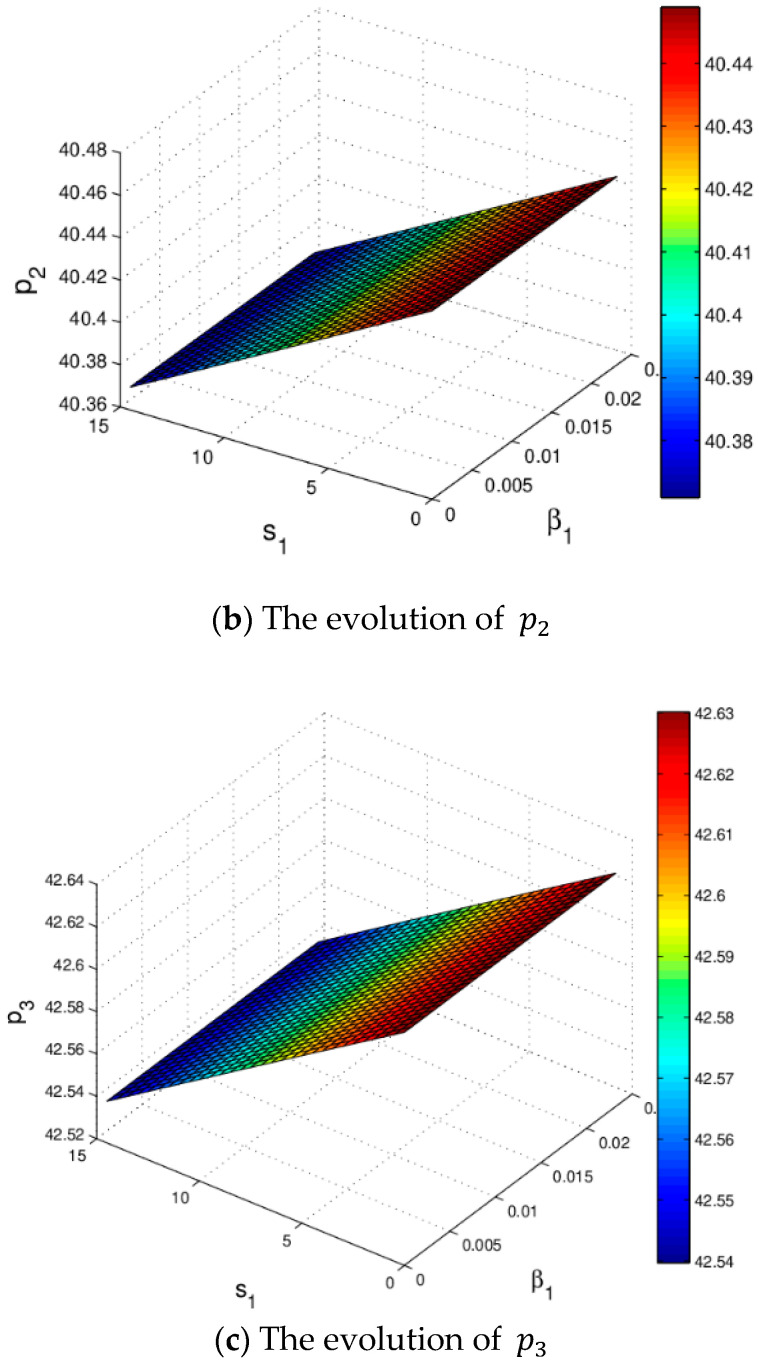
The evolution of the system (11) with respect to $\beta_{1}\;\mathrm{and}\;s_{1}$.

Figure 18 shows the stable region of $\beta_{1}\;\mathrm{and}\;\beta_{2}$ with change of $s_{1}$, we can see that the stable region of the system (11) decreases with increase of $s_{1}$. Figure 19 give the price evolution of the system (11) with change of $s_{1}\;\mathrm{and}\;\beta_{1}$. In Figure 19a, the system (11) occurs the first bifurcation when $\beta_{1}=0.0177$ and $s_{1}=0$; occurs the first bifurcation when $\beta_{1}=0.0165$ and $s_{1}=15$. We can see that the higher the service level is, the easier the bifurcation occurs. With $s_{1}$ increasing, entropy is increasing gradually and the system falls into chaos. What is more, it can easily find that the increase of $s_{1}$ has little effect on the prices of manufacturer, but greatly improves the price of the retailer.

Above all, we can get that service level has obvious impact on the dynamics system (11). The high service level decreases the stability domain and increases system entropy, results the system (11) falls into chaos earlier. Besides, the service level of retailer has great influence on the price of itself. 

#### 4.3.4. The Influence of Service Level on System Profit 

**Figure 20:**
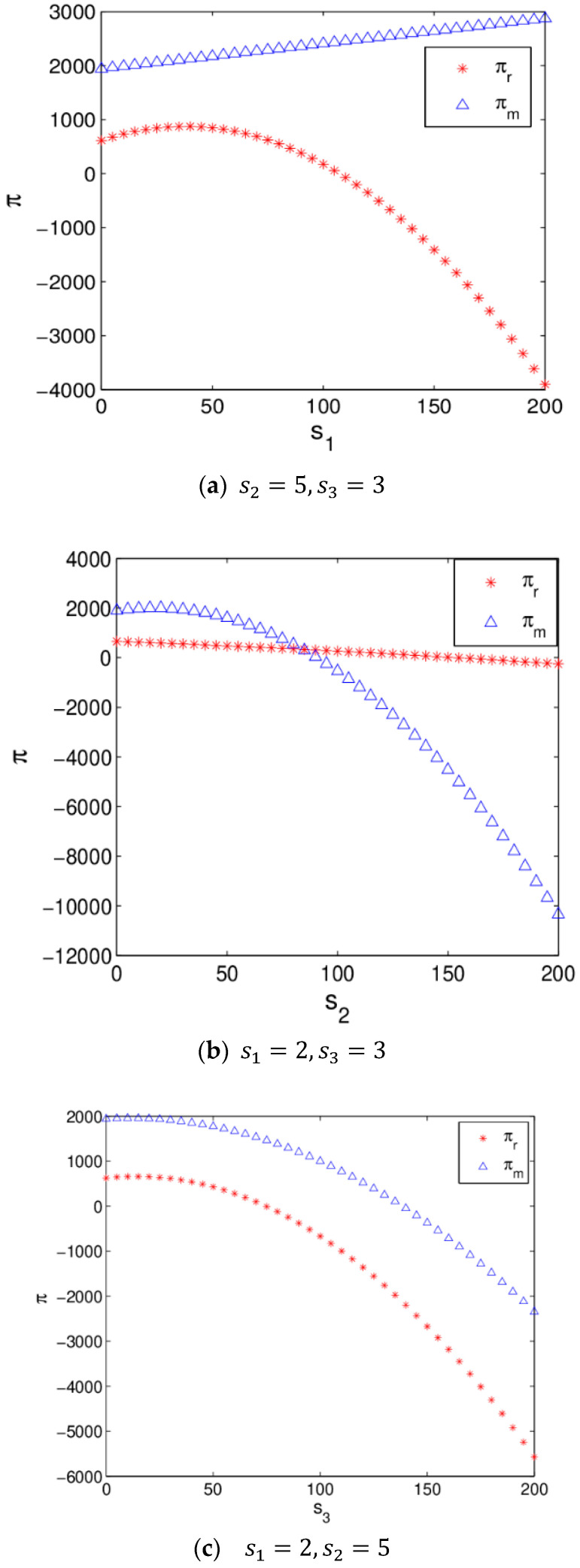
The profit evolution respect to service level.

Figure 20 shows the profit evolution of the manufacturer and retailer with the change of service level. The manufacturer’s profit increases and the retailer’s profit increases first and then decreases with $s_{1}$ increasing in Figure 20a. In Figure 20b, the profit of the retailer decreases and the manufacturer’s profit increases first and then decreases as $s_{2}$ increases, which is consistent with reality. Figure 20c shows that the profits of the manufacturer and retailer decrease as $s_{3}$ increases, which is not conducive to the realization of profit maximization of the manufacturer and retailer by improving the service level of OSC, because consumers are not sensitive to the services of OSC and the service cost rises with improving service level, so the manufacturer should be careful when making service decisions. 

**Figure 21:**
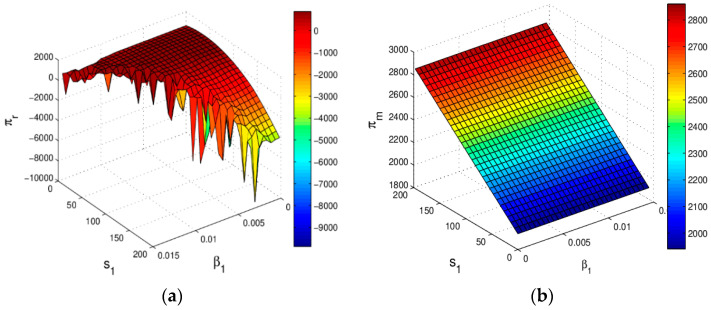
The change of profit respect to $\beta_{1}\;\mathrm{and}\;s_{1}$ when $\beta_{2}=0.015\;\mathrm{and}\;\beta_{3}=0.03$.

Figure 21 shows the profit evolution of the manufacturer and retailer vary with $\beta_{1}$ and $s_{1}$ changing. When $\beta_{1}$ and $s_{1}$ are maintained at an appropriate range, the system profit is stable. With $\beta_{1}$ increasing, the profit of the retailer becomes unpredictable and finally gets into chaos in Figure 21a. It can be seen clearly that system effectiveness in chaos is obviously lower than that in the stable state. Under the Stackelberg game, the stability of manufacturer’s profit is not affected by $s_{1}$ which is showed in Figure 21a. In the market competition, the retailer should keep the price adjustment speed and service level in the appropriate range, which is conducive to the stability and improvement of the retailer’s profit.

## 5. Conclusions

In this paper, a multi-channel supply chain that consists of a manufacturer and a retailer is studied. Considering channel service and channel cooperation, we propose a Nash dynamic game model and a Stackelberg dynamic game model based on the marginal profit and BRE rule. The complexity characteristics of the two dynamic game models are investigated. We research the effect of the price adjustment speed on the system stability and analyze the influence of service level on system’s profit and the stability of system. From the complexity analysis and the experimental designs, we can find some important conclusions that:

(1) the greater the service level and profit distribution rate are, the smaller the stability domain of the system is;
(2) with the price adjustment speed gradually increasing, the price system gets unstable and finally becomes chaotic;
(3) when the manufacturer or the retailer keeps service level in the appropriate value which is conducive to maximizing her/his profits;
(4) in Nash game model, the stability of the system weakens than that in the Stackelberg game model.

In spite of the contribution that this paper has offered to the managers, some limitations still exist in this paper, this paper only consider the influence of the channel cooperation and service on the multi-channel supply chain, besides, what impact the risk attitude of decision and manufacturer’s innovation input have on the supply chain system? Next, we will put interesting on them.
